# Pandemic Influenza: Risk of Multiple Introductions and the Need to Prepare for Them

**DOI:** 10.1371/journal.pmed.0030135

**Published:** 2006-02-21

**Authors:** Christina E Mills, James M Robins, Carl T Bergstrom, Marc Lipsitch

## Abstract

Containing an emerging influenza H5N1 pandemic in its earliest stages may be feasible, but containing multiple introductions of a pandemic-capable strain would be more difficult. Mills and colleagues argue that multiple introductions are likely, especially if risk of a pandemic is high.

The World Health Organization recently stated the following: “Since 2003, the world has moved closer to a[n] [influenza] pandemic than at any time since 1968” [ 1]. Influenza A (H5N1) viruses have reached high prevalence in both domesticated and wild birds in several parts of Asia [ 2]; the virus has spread over an area ranging from Romania to Indonesia, possibly carried over this distance by migratory waterfowl [ 3]. Over 160 human cases, about half of them fatal, have occurred [ 4], from Indonesia to Turkey. These trends suggest an increasing risk that the virus may acquire the ability to transmit efficiently from human to human, equipping it to cause a new pandemic.

Multiple measures are required to prevent such a pandemic and, if these fail, to detect it, check its spread, and mitigate its effects. Attention has recently focused on the possibility of containing a pandemic in its earliest phases at its source, using antiviral drugs, before global spread occurs. Two recent studies [ 5, 6] concluded that containment might work, under certain assumptions. First, the strain must be only moderately transmissible, with a basic reproductive number of 1.8 or less. In other words, this means that containment is likely to work only if each individual infected with the pandemic strain infects an average of 1.8 or fewer additional individuals. If such a strain emerged, the models suggest, containment would require that several further conditions also be met: the emerging pandemic is detected within the first 20 cases [ 6] or 7–21 days [ 5]; thereafter, antiviral prophylaxis is delivered efficiently to a geographic area (e.g., to an area with a ten kilometer radius) around 90% of clinical cases within two days of symptom onset; the strain remains susceptible to oseltamivir (a growing concern following reports of partially and fully resistant strains among recent H5N1 cases [ 7, 8]); and adequate antiviral supplies (between 100,000 and 3 million courses) are available.

Even if these conditions are met and the next introduction of a pandemic-capable strain is contained, a containment policy alone is unlikely to prevent a pandemic entirely. We argue here that if a single introduction of a pandemic-capable strain is expected, multiple introductions should also be expected. Each containment effort would likely be more difficult than the last as manpower, antiviral stockpiles, and other scarce resources become depleted. Even if each successive containment effort is no more difficult than its predecessor, the chance of at least one failure increases with the number of introductions. At best, a containment policy will only postpone the emergence of a pandemic, “buying time” to prepare for its effects.

In this article, we consider the risk of multiple introductions and its implications for pandemic planning.

## How Likely Are Multiple Introductions?

Since the last pandemic nearly 40 years ago, we have observed dramatic changes in social and ecological factors thought to facilitate emergence of a pandemic-capable strain. Surging human and bird populations in Asia have increased the frequency of contact between birds and humans—and these changes might facilitate emergence by permitting “crossing over” of a mutated avian influenza to humans, or by allowing human and avian influenzas to reassort in the same animal host. Already, the cases of human infection with avian influenzas are mounting. From 2003 to February 2, 2006, there have been 161 documented cases of and 86 deaths due to highly pathogenic H5N1 avian influenza infections in humans [ 4]. Serologic evidence indicates that humans have been infected with many types of avian influenzas [ 9], not just the easily ascertained, highly pathogenic H5N1. Though each human case of avian influenza, acquired from a bird exposure, has a low probability of creating a virus strain capable of pandemic spread, H5N1 influenza has probably spread from human to human within one family [ 10], and several additional cases may also have involved human-to-human transmission [ 11]. In considering the overall risk that a pandemic strain will emerge in a particular time period, the relevant figure is not the risk that a single infection will lead to a pandemic, but the probability that any of the human or animal cases occurring over that period will give rise to a strain capable of sustained human-to-human transmission and that the strain will begin spreading in people.

If a pandemic-capable H5N1 strain emerges once in humans and is contained, is it likely to emerge again? One might hope to look to history for evidence on this question. Genetic evidence of multiple reassortment events or mutations early in the 1968 H3N2 pandemic [ 12] is consistent with the possibility of multiple introductions, but does not demonstrate this conclusively. We are not aware of such evidence for other pandemics, none of which were contained. However, one might expect that if multiple introductions did occur but none were contained, the evidence for such introductions might be difficult to detect because of depletion of susceptible hosts by the first uncontained introduction, competitive exclusion by the most transmissible of the introduced strains, or other factors.

**Figure 1:**
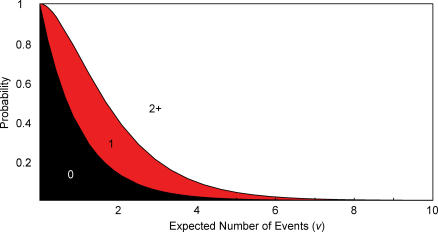
Probabilities of Zero, One, or Two or More Events (Introductions of a Pandemic-Capable Strain) When the Expected Number of Events Ranges from Zero to Ten, under a Constant Hazard (Poisson Distribution) Note that if the expected number of events *(v)* is more than 1.26, then it is more likely that two or more events will occur than that exactly one event will occur.

When a strain emerges in one locale, the fundamental processes of bird-to-bird and bird-to-human transmission that generate introductions need not change elsewhere. Thus, a single emergence may neither increase nor decrease the chance that another emergence will happen the next day or week, nearby or in another country. Under the assumption that the risk (or hazard) of emergence remains constant over time, if one introduction is likely in a given period, then multiple introductions are also likely. Figure 1 shows the relationship between the risks of zero, one, or more than one introduction under the constant hazard assumption (which generates a Poisson distribution of introductions in a given period). As the expected number of introductions in a given period, which we call *v*, increases from zero, the likelihood of a single introduction also rises (along with the likelihood of more than one); as it increases further, a single introduction becomes less likely and multiple introductions more likely.

More plausibly, one might expect that the first introduction is likely to occur because something has changed in the virus circulating in birds: the avian strain has increased in prevalence in a particular area [ 2, 3, 13], spread to new geographic areas [ 2], or evolved in such a way that it is better suited to human transmission [ 14]. This last scenario—that the virus could evolve within birds to a form that is capable of pandemic transmission in humans—has recently become more plausible given evidence that the 1918 pandemic H1N1 strain was transferred from birds to humans without reassortment [ 15]. If the hazard of introductions depends on any of these factors, then observing a single introduction would signal that the risk of further introductions had increased substantially, and introductions would be clustered. Under either assumption, if we believe an introduction is likely, then we must also expect a high risk of multiple introductions.

## Quantifying the Benefit of a Containment Policy Given the Possibility of Multiple Introductions

The benefit of a containment policy can be defined as the expected increase in the time to a pandemic achieved by the policy. The realized benefits—in terms of lives and dollars saved—will depend heavily on the available resources and technology, the level of pandemic preparedness, and the timeliness of action. Importantly, the window of opportunity provided by postponing a pandemic must be wide enough to successfully implement procedures that reduce the risk of a pandemic (e.g., poultry culling and prevention of bird–human contact), to produce effective vaccines (sufficient for those at risk of severe outcomes, at a minimum, and/or sufficient for blocking sustained transmission) and to prepare populations and their health systems for the expected onslaught of influenza cases.

The expected gain in time to a pandemic *(G)* depends on several parameters (Text S1 contains additional supporting methodology and results). The first parameter is the expected time to introduction of a pandemic-capable strain *(T)*. In the simplest case, where the risk of introduction at each moment (the hazard) remains fixed, the expected time to introduction *(T)* is 1/λ, where λ is the rate at which we expect pandemic-capable strains to be introduced. Secondly, the gain in time from a containment policy depends on whether such a policy equipped us for an indefinite number of containment attempts, each with the same probability of success (a probably unachievable extreme), or whether, at the other extreme, only the first introduction can be contained. A third factor affecting the gain is whether we believe λ, the hazard of introduction of a pandemic strain, is constant over time, or whether λ is increasing due to changes in factors such as the genetic make-up of avian influenza strains, the contact rate between humans and birds, or the number of infected birds available to contact humans.

**Figure 2:**
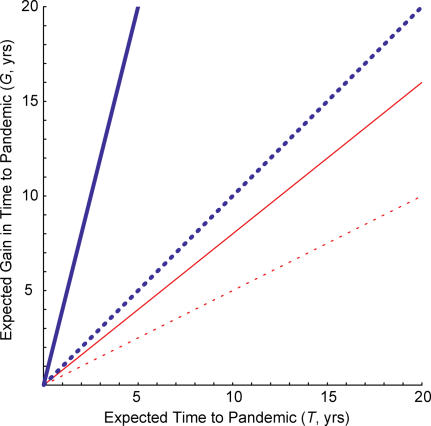
Expected Gain in Time to a Pandemic *(G)* of a Containment Policy as a Function of the Expected Time to Introduction *(T)* of a Pandemic-Capable Strain Either infinite containment attempts (blue) are possible or only a single containment attempt (red) is possible, and we assume an optimistic (80%, solid) or pessimistic (50%, dashed) probability of success for each containment. *G* is defined as the difference in the expected time to a pandemic under the status quo and the containment policy. The Poisson hazard of introduction of pandemic-capable strains *(λ)* is the reciprocal of the expected time to introduction *(T)*. The results are identical if the hazard of introduction is fixed but unknown.

Figure 2 considers the simplest case, where the hazard λ remains fixed over time, and shows how the expected gain in time to a pandemic depends on the expected time to introduction *(T* = 1/λ *)*, the ability to contain multiple pandemic-capable strains, and the success probability for each containment attempt *(c)*. The solid blue line shows the most optimistic assumptions—unlimited capacity to respond to multiple introductions and an 80% success probability of containment ( Figure 2, solid blue line). The most effective containment policy considered in Ferguson et al. [ 6] (i.e., “drug sparing” antiviral prophylaxis combined with school closing and area quarantine) had an efficacy of 90% or more against an influenza strain that is less transmissible than previous pandemic influenza strains [ 16, 17], conditional on the assumptions of the model (adequate and timely response, introduction in a rural area, oseltamivir sensitivity, etc.) being met. We consider 80% success probability to be optimistic after taking into account the risk that one or more of these conditions would not be met in any given introduction.

In these circumstances, the expected increase in the time to a pandemic is greater than the expected time to a pandemic in the absence of containment. For example, if the expected time to a pandemic is one year in the absence of containment, the expected gain of an 80% successful containment policy is four years, assuming that containment can be implemented an indefinite number of times. However, if the probability of successful containments is less than or equal to 50% ( Figure 2, dashed blue line) or if only the first pandemic-capable strain can be contained, regardless of the success probability ( Figure 2, red lines), the expected gain will be less than or equal to the expected time to a pandemic. If the expected waiting time until the occurrence of a pandemic is one year, the expected gain of a 50% successful “one shot” containment policy would be only six months. Thus, under most circumstances, a containment policy will (in expectation) “buy” no more than the expected time to the pandemic. If the expected waiting time until the occurrence of a pandemic is five years, under realistic assumptions, the expected gain might be on the order of one or two years. Without prior planning and investment, this gain would be insufficient to effect change.

## Containment Provides Smaller Benefits if the Hazard of Introduction Is Increasing

Thus far, we have assumed a fixed hazard of introduction. A number of social, ecological, or evolutionary changes in the virus could cause escalation of the hazard over time, either in an abrupt or in a gradual fashion. First, as the avian epidemic progresses, the virus could undergo genetic change in birds, with the side consequence of more effective spread in humans. Even relatively small changes that allow only short chains of chance transmission in humans might dramatically increase the probability of disease emergence by allowing the virus time to evolve to human transmission [ 18]. In addition to genetic change in the virus population circulating in birds, simple ecological changes, such as increased prevalence of the infection in birds, could also lead to gradual increases in the hazard of introduction with time.

**Figure 3:**
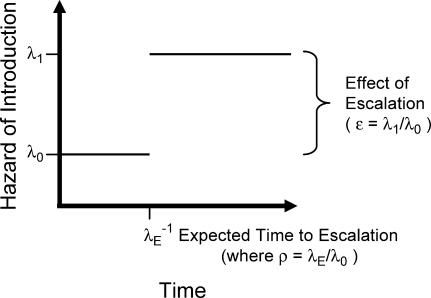
Schematic Diagram of the Escalating Hazard of Introduction Model with Time on the Horizontal Axis and Hazard of Introduction of a Pandemic-Capable Strain on the Vertical Axis The initial hazard of introduction (λ _*0*_) increases to a higher hazard of introduction (λ _*1*_) at a rate determined by the hazard of escalation (λ _*E*_). The expected time of escalation is the reciprocal of the hazard of escalation. The effect of escalation on the hazard of introduction is measured by the ratio of the final to the initial hazards of introduction *(ε =* λ _*1*_/λ _*0*_).

The benefits of containment decrease if we face a growing hazard of introduction, regardless of whether this growth is abrupt or gradual (see Text S1 for details). Suppose that we believe the hazard of introduction of a pandemic-capable strain is at some level, λ _*0*_, now but may escalate to a higher level, λ _*1*_, in the future ( Figure 3). We call the ratio of these rates (λ _*1*_/λ _0_) the effect of escalation (ε *)*, where escalation may be caused by any number of genetic, environmental, and social changes that might precipitate a relatively rapid increase in the rate of introduction of a pandemic-capable strain. Escalation itself is assumed to occur with some hazard rate (λ _*E*_). The relative magnitude of the escalation hazard is the ratio of the hazard of escalation to the initial hazard of introduction (λ _*E*_/λ _*0*_), which we call ρ (see Text S1).

**Figure 4:**
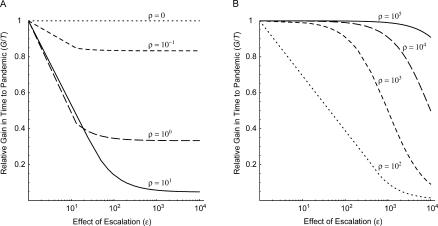
Relative Gain in Time to a Pandemic *(G/T)* as a Function of the Effect of Escalation on the Hazard of Introduction *(ε = λ _1_/λ _0_) *, Assuming Infinite Containment Attempts with 50% Success Probability Are Possible Each curve corresponds to a 10-fold increase in the magnitude of the escalation hazard relative to the initial hazard of introduction *(ρ = λ _E_/λ _0_) *, ranging from 0.1 to 10 (A, short-dashed, long-dashed, and solid lines), and from 100 to 100,000 (B, dotted, short-dashed, long-dashed, and solid lines). When the relative magnitude of the escalation hazard is zero (A, dotted line), the relative gain in time to a pandemic is identical to the simple Poisson process in Figure 2.

Figure 4 shows how the gain in time to a pandemic depends on the expected time to introduction. We plot the relative gain in time to a pandemic *(G/T)*, defined as the expected gain *(G)* divided by the expected time to a pandemic in the absence of containment *(T)*, against the effect of escalation (ε), assuming a range of relative magnitudes of the escalation hazard (ρ) and a 50%–success probability containment policy that can be implemented an infinite number of times. In the limiting case where there is no escalation (i.e., ρ = 0), the results are identical to Figure 2 for a 50%–success probability infinite-attempts containment policy: the slope of the expected time to introduction *(T)* versus expected gain in time to introduction *(G)* curve ( Figure 2, dashed red line) is equal to the relative gain under the same containment policy *(G/T*, Figure 4A, dotted line). As the relative magnitude of the escalation hazard increases ( Figure 4A, short-dashed, long-dashed, and solid line), the relative gain falls below one. The depression in the relative gain is greater when the effect of escalation is large and when the relative magnitude of the escalation hazard is high because escalation would be expected in the foreseeable future, though it may not occur before the first introduction. When the relative magnitude of the escalation hazard is extremely high ( Figure 4B), escalation is expected prior to any introduction, and the relative gain approaches one. Similar results are obtained for a containment policy when only one containment effort is achievable (with the relative gain bounded by the success probability *[c]* and for the situation when the growth of the hazard of introduction is gradual, see Text S1). Thus, the relative gain of a containment policy is maximized for a limited set of parameter values: when the hazard of escalation is extremely slow or extremely fast relative to the initial hazard of introduction, and when the effect of escalation is quite small. Even under these assumptions, however, the relative gain does not exceed one.

## Conclusions and Policy Implications

We conclude that the benefit of a strategy to contain pandemic influenza at its source will be to postpone the time to the next pandemic, not to prevent a pandemic from ever occurring. If at least one containment attempt of an introduced pandemic-capable strain will be necessary, it is likely that multiple containment attempts will be necessary. Richard Danzig has made a similar point about preparations for bioterrorist events. Because terrorists using biological agents could “reload” with relative ease and strike multiple times in multiple locations, Danzig cautions that we should “plan to defend against a campaign, not an isolated attack” [ 19].

Even if we had the capacity (i.e., stockpiles, resources, and manpower) to undertake multiple containment attempts, each introduction of a pandemic-capable strain offers a new chance for containment to fail, and subsequent introductions would likely be harder to contain than the first. Thus, containment is likely to “buy” time, but the amount of time will depend on the capacity for multiple containment attempts and the success probability of any given containment attempt, among other factors.

For most realistic scenarios, containment provides only a small expected gain in time to a pandemic. The expected gain is generally less than the expected time to the next pandemic. Because of its potentially limited impact, we therefore conclude that containment should not form the centerpiece of a pandemic control policy. Moreover, to maximize the benefits of any delay achieved by containment, any containment plan should include an additional action plan to be implemented rapidly and concurrently with the first containment attempt. Central to this action plan should be a series of measures to drastically reduce the risk of introduction of a pandemic-capable strain, including widespread culling of exposed or potentially exposed poultry and changes in agricultural practices to reduce ongoing risk of exposure [ 20]. Significant poultry culling in and around the site of introduction would shrink the animal reservoir population and would limit bird–human contact. Culling in more distant sites would be warranted if the pandemic-capable strain or related strains were widely distributed. Vaccination of avian populations might be an option in areas where the risk is less, or where culling is not practical. Such measures, the most extreme of which may not be politically or economically viable at present, would take on additional urgency and perhaps become possible following the introduction, and successful containment, of a pandemic-capable strain.

Preventing further introductions by reducing the hazard of introduction would enhance the benefits of a containment policy in two ways. As the hazard of introduction decreases and the expected time between introductions increases, the gain in time to a pandemic would increase. Furthermore, the number and success probability of future containment attempts would increase, since there would be additional time to replenish resources and improve containment plans.

The first successful containment attempt must also trigger accelerated preparations for an uncontained pandemic. It is especially critical to accelerate development, testing, and licensing of vaccines that can be produced in sufficient quantities to immunize whole populations in the event of a pandemic. In addition, it is imperative that preparations are made at the national, state, and city level to cope with the health, social, and economic effects of a severe pandemic. These and other strategies to increase preparedness require a substantial lead time before they can substantially affect the impact of a pandemic.

Of course, we might never have the opportunity to implement any of these responses; the first containment attempt might fail. Therefore, if we identify measures that would be useful following a successful containment effort, there is likely to be a strong case for implementing those measures now, rather than waiting until the first introduction of a pandemic strain.

Containment at the source of introduction should thus be considered only one part of a multipronged strategy that includes vaccine development, improved surveillance and public health response capabilities, deployment of measures to reduce the likelihood of introduction (especially after the first introduction), and detailed planning for measures to mitigate the health, social, and economic impact of a pandemic. In emphasizing the need for a multifaceted approach, we echo the recommendations of international organizations [ 1, 20] and of the original modeling studies of containment, which emphasized the need for better surveillance [ 6] and the value of vaccination [ 5] in concert with containment efforts.

If preparation for a potential influenza pandemic is an important public health goal, as most experts agree it should be, substantial investments and planning are needed to maximize the benefits of a containment policy and to minimize the deleterious consequences of the next influenza pandemic.

## Supporting Information

Text S1 Additional Supporting Methodology and Results (273 KB PDF).

Alternative Language Abstract Translation of the Abstract into Thai by Pantipa Rakkuson, Warakan Poomkumarn, and Christina E. Mills (35 KB PDF).
