# Supplementary material for: Pandemic Influenza: Risk of Multiple Introductions and the Need to Prepare for Them
Source: PLoS Med. 2006 Feb 21;3(6):e135. doi: 10.1371/journal.pmed.0030135 (PMC1370924; doi:10.1371/journal.pmed.0030135)
Supplement: Supplementary file 1 [file pmed.0030135.sd001.pdf]

SUPPORTING INFORMATION to

PANDEMIC INFLUENZA: RISK OF MULTIPLE INTRODUCTIONS AND THE NEED TO PREPARE FOR THEM

Christina E. Mills, James M. Robins, Carl T. Bergstrom, Marc Lipsitch

I. Methods for Calculating Benefit

A. Models for the Hazard of Introduction of Pandemic-Capable Strains

The main text considers models in which of the hazard of introduction is constant, and models in which the hazard of introduction increases over time. Below we present in detail two constant hazard models and two increasing hazard models.

1. Constant Hazard of Introduction Models

a) Constant Hazard of Introduction Model with a Known Hazard of Introduction

In this model, we assume that the introduction of a pandemic-capable strain is a homogeneous Poisson process with hazard λ beginning at the present time (defined as zero). When the hazard is fixed, the distribution of the number of introductions in a given time frame is Poisson and the distribution of the times to event is exponentially distributed.

b) Constant Hazard of Introduction Model with an Unknown Hazard of Introduction

We also consider a more general form of the constant hazard model where the hazard of introduction is unknown and distributed with some probability density function $f_\lambda(\lambda)$. As is shown below, the benefit of containment, as quantified by the relative gain (G/T), does not depend on $f_\lambda(\lambda)$. Thus the results for this model are equivalent to those for the constant hazard of introduction model with a known hazard of introduction and are not discussed further.

When the unknown hazard of introduction is Gamma distributed, the number of introductions in a given time frame will be distributed as a negative binomial. This distribution describes the "clustered" model discussed in the text. Supporting Figure 1 shows the probabilities of zero, one, or two or more introductions of a pandemic-capable strain when the expected number of events ranges from 0 to 10 for a negative binomial distribution. This distribution is different from the Poisson distribution in Figure 1 in two ways. First, the threshold at which the probability of greater than one introduction of a pandemic-capable strain is greater than the probability of exactly one event is one. This threshold is slightly higher (1.26) for a Poisson distribution. Second, the probability of exactly one event for the negative binomial distribution is greater than that for the Poisson distribution for large expected number of events.

2. Escalating Hazard of Introduction Models

a) Stochastically Timed Jump in the Hazard of Introduction Model

In this model, the introduction of a pandemic-capable strain is a Poisson process with hazard λ_0 over the time interval $(0, t_E)$, and a higher hazard λ_1 over the time interval (t_E, ∞). At time t_E there is an increase in the hazard of introduction. For example, this increase might be due to evolution of the virus circulating in birds so that it is genetically "closer" to being equipped for human-to-human spread. The time to escalation is exponentially distributed with hazard λ_E and expected time to escalation λ_E^{-1}. The effect of escalation ε is the ratio between the post-and pre-escalation hazards of introduction.

The relative magnitude of the hazard of escalation ρ is the ratio of the hazard of escalation to the initial hazard of introduction.

b) Deterministic Continuous Increase in the Hazard of Introduction Model
In this model, the hazard of introduction is a deterministic function determined by the parameter r and the initial hazard of introduction λ_0. This function is either (i) an exponential function so that the hazard rate is given by $\lambda(t) = \lambda_0 e^{rt}$ or (ii) a linear function of time with hazard rate $\lambda(t) = \lambda_0 rt$. For example, this continuous increase might be due to an increase in the prevalence of the virus in birds.

B. Calculation of the Benefit of a Containment Policy
As stated in the text, we define the benefit of a containment policy as the expected gain (G) in the time to a pandemic under a policy to contain a pandemic at its source. The relative gain is defined as the ratio of the expected gain (G) divided by the expected time to a pandemic under the status quo (T). The gain and relative gain are calculated as follows

G = E[g]
G/T = E[g]/ E[t]

where t is the time to a pandemic under the status quo, and g is the difference in time to a pandemic under the status quo and the time to a pandemic under the containment policy. Below we calculate the benefit of a containment policy assuming each of the models described above. We present the calculations in a different order because the constant hazard of introduction model is a special case of the stochastically timed jump in the hazard of introduction model.

1. Stochastically Timed Jump in the Hazard of Introduction Model
The probability density function (PDF) for the time to first introduction ($f_t(t)$) can be written as

$$f_t(t) = \int_0^t f(t - t_E \mid \lambda_1)(1 - F(t_E \mid \lambda_0)) f(t_E \mid \lambda_E) dt_E + f(t \mid \lambda_0)(1 - F(t \mid \lambda_E))$$

where $f(x|\lambda)$ and $F(x|\lambda)$ are the PDF and cumulative distribution function, respectively, of an exponentially distributed random variable with rate parameter λ, evaluated at x. The first and second terms are the probability of t if evolution occurs before and after introduction, respectively. The PDF and expected value for the time to first introduction are

$$f_t(t) = \left(\lambda_0 e^{-\lambda_0 t}\right)\left(e^{-\lambda_E t}\right) + \left(\lambda_1 e^{-\lambda_1 t}\right)\left(1 - e^{-(\lambda_E + \lambda_0 - \lambda_1)t}\right)\left(\frac{\lambda_E}{\lambda_E + \lambda_0 - \lambda_1}\right)$$

$$T = E[t] = \frac{\lambda_1 + \lambda_E}{\lambda_1(\lambda_0 + \lambda_E)}$$

The PDF for the gain in time to a pandemic ($f_g(g)$) assuming a single containment attempt with 100% success probability can be written as

$$f_g(g) = f_t(g)\int_0^{\infty} f(t_E \mid \lambda_E)F(t_E \mid \lambda_0)dt_e$$
$$+ f(g \mid \lambda_1)\int_0^{\infty}(1 - F(t_E \mid \lambda_0))f(t_E \mid \lambda_E)\int_{t_E}^{\infty} f(t - t_E \mid \lambda_1)dtdt_e$$

where $f_t(g)$ is the PDF for the time to first introduction (calculated above) evaluated at g. The first term and second terms are the probability of g if the first introduction occurs before and after escalation, respectively. The first term is the product of three quantities integrated over all possible times of escalation: the probability that the first introduction will occur before escalation, the probability of escalation occurring at time t_E, and the probability of g given that the first introduction occurs before escalation. Note that the gain given that escalation does not occur before the first introduction has the same PDF as the time to the first introduction. The second term is the product of four quantities integrated over the time of first introduction and the time of escalation: the probability the first introduction will occur t-t_E after escalation, the probability that the first introduction will not occur by t_E, the probability of escalation occurring at time t_E, and the probability of g given that the first introduction occurs after escalation. Note that the gain given that escalation does occur before the first introduction has the same PDF as the time to introduction when the hazard is constant.

The PDF and expected value for the gain are

$$f_g(g) = \frac{\left(e^{-(\lambda_E+\lambda_0+2\lambda_1)g}\right)\left(\lambda_0(\lambda_0 - \lambda_1)(\lambda_E + \lambda_0)e^{\lambda_1 g} + \lambda_1\lambda_E(\lambda_E + 2\lambda_0 - \lambda_1)e^{(\lambda_E+\lambda_0)g}\right)}{(\lambda_E + \lambda_0 - \lambda_1)(\lambda_E + \lambda_0)}$$
$$G = E[g] = \frac{\lambda_0\lambda_1 + 2\lambda_0\lambda_E + \lambda_E^2}{\lambda_1(\lambda_0 + \lambda_E)^2}$$

The expected gain for a single containment attempt with success probability c is calculated by scaling the expected gain by c. If infinite containment attempts with success probability c are possible, the expected gain is calculated as the difference between the time to first introduction with hazards of introduction scaled by (1-c) and the time to first introduction. The relative gain for a containment attempt is then the ratio between the expected gain under that policy and the time to first event. The relative gain formulas are written in terms of the effect of escalation ($\varepsilon = \lambda_1 / \lambda_0$) and the relative magnitude of the hazard of escalation ($\rho = \lambda_E / \lambda_0$).

G/T for a single containment attempt: $c\left(\frac{\varepsilon + 2\rho + \rho^2}{(1+\rho)(\varepsilon + \rho)}\right)$

G/T for infinite containment attempts: $\left(\frac{c}{1-c}\right)\left(\frac{\varepsilon + 2\rho + \rho^2 - c(\varepsilon + \rho)}{\varepsilon + (1+\varepsilon)\rho + \rho^2 - c(\varepsilon + \rho)}\right)$

2. Constant Hazard Model with a Known Hazard of Introduction
To obtain the PDFs and expected values for *t* and *g* assuming the constant hazard model with a known hazard of introduction, we set the hazard of escalation to zero and the initial hazard to λ.

$$f_t(t) = \lambda e^{-\lambda t}$$
$$T = E[t] = \frac{1}{\lambda}$$

$$f_g(g) = \lambda e^{-\lambda g}$$
$$G = E[g] = \frac{1}{\lambda}$$

The relative gain formulas are then *c* for a single containment attempt and *c*/(1-*c*) for infinite containment attempts.

3. Constant Hazard Model with an Unknown Hazard of Introduction
If the hazard of introduction λ is unknown with PDF $f_\lambda(\lambda)$, the PDFs and expected values for *t* and *g* are

$$f_t(t) = \int_0^\infty \lambda e^{-\lambda t} f_\lambda(\lambda) d\lambda$$
$$T = E[t] = E\left[\frac{1}{\lambda}\right]$$
$$f_g(g) = \int_0^\infty \lambda e^{-\lambda g} f_\lambda(\lambda) d\lambda$$
$$G = E[g] = E\left[\frac{1}{\lambda}\right].$$

The relative gain formulas are then *c* for a single containment attempt and *c*/(1-*c*) for infinite containment attempts. The relative gain is thus the same regardless of whether the hazard is known or unknown.

4. Deterministic Continuous Increase in the Hazard of Introduction Model
Following Taylor and Karlin (1998; p.37) we can write the cumulative density function (CDF) for the time of the first event after time 0 as a function of the hazard rate $\lambda(t)$:

$$F(0,t) = 1 - e^{-\int_0^t \lambda(x) dx}$$

For exponential model where $\lambda(0) = \lambda_0$, this corresponds to $F(0,t) = 1 - e^{(1-e^{rt})\lambda_0 / r}$
The PDF for the first event after time 0 is the time derivative of the CDF:

$$f(0,t) = \lambda_0 e^{rt + (\lambda_0 / r)(1 - e^{rt})} \; .$$

The mean time of the first event is therefore $-e^{\lambda_0/r}Ei[-\lambda_0/r]/r$ where $Ei[x]=-\int_{-x}^{\infty}\frac{e^{-t}}{t}dt$ is the exponential integral function.

The PDF of occurrence times for the second event is given by the convolution integral

$$\int_0^t f(0,x)f(x,t)dx$$

For the exponential increase model, this is

$$f(t)=-e^{rt+(1-e^{rt})(\lambda_0/r)}(1-e^{rt})\lambda_0^{\ 2}/r$$

The expected gain (i.e. the mean occurrence time of the second event less the mean occurrence time of the first event) is $1/r+e^{\lambda_0/r}\lambda_0 Ei[-\lambda_0/r]/r^2$.

The relative gain (i.e. the ratio of the expected gain to the mean occurrence time of the first event) is $-\frac{1}{\phi}-\frac{e^{-\frac{1}{\phi}}}{Ei[-\frac{1}{\phi}]}$ where ϕ is the ratio $\frac{r}{\lambda_0}$.

For the linear increase model, the hazard begins at λ_0 and increases at a rate r per unit time. Then the CDF and PDF of the first event are $F(0,t)=1-e^{-rt^2\lambda_0/2}$ and $f(0,t)=(\lambda_0+rt)e^{-\lambda_0 t-rt^2/2}$ respectively. The mean time of first event is $\exp\left(\frac{\lambda_0^2}{2r}\right)\sqrt{\frac{\pi}{2r}}\left(1-\mathrm{erf}\left[\frac{\lambda_0}{\sqrt{2r}}\right]\right)$, where $\mathrm{erf}(x)=\frac{2}{\sqrt{\pi}}\int_0^x e^{-t^2}dt$. Proceeding as above, we can compute the PDF of the second event time: $\frac{t(rt+\lambda_0)(rt+2\lambda_0)}{2}\exp\left(\frac{1}{2}t[rt+2\lambda_0]\right)$ which gives us a mean time of the second event of $\frac{1}{2}\left(\frac{\lambda_0}{r}+\frac{\exp\left(\frac{\lambda_0^2}{2r}\right)\sqrt{\frac{\pi}{2}}(3r-\lambda_0^2)(1-\mathrm{erf}[\lambda_0/\sqrt{2r}])}{r^{3/2}}\right)$. The relative gain for this model is therefore $\frac{G}{T}=\frac{1}{2}-\frac{x}{2}+\frac{\exp(-x/2)\sqrt{x}}{\sqrt{2\pi}\left(1-\mathrm{erf}\left[\sqrt{\frac{x}{2}}\right]\right)}$, where $x=\lambda_0^2/r$

5. Table of Formulas for *T, G* and *G/T*.

**Escalating Hazard of Introduction Model**		
Measure	Single Containment Attempt	Infinite Containment Attempts
T	$\frac{\lambda_1+\lambda_E}{\lambda_1(\lambda_0+\lambda_E)}$	$\frac{\lambda_1+\lambda_E}{\lambda_1(\lambda_0+\lambda_E)}$
G	$c\left(\frac{\lambda_0\lambda_1+2\lambda_0\lambda_E+\lambda_E^2}{\lambda_1(\lambda_0+\lambda_E)^2}\right)$	$\frac{(1-c)\lambda_1+\lambda_E}{(1-c)\lambda_1((1-c)\lambda_0+\lambda_E)}-\frac{\lambda_1+\lambda_E}{\lambda_1(\lambda_0+\lambda_E)}$
G/T	$c\left(\frac{\varepsilon+2\rho+\rho^2}{(1+\rho)(\varepsilon+\rho)}\right)$	$\left(\frac{c}{1-c}\right)\left(\frac{\varepsilon+2\rho+\rho^2-c(\varepsilon+\rho)}{\varepsilon+(1+\varepsilon)\rho+\rho^2-c(\varepsilon+\rho)}\right)$
**Constant Hazard of Introduction Model**		
Measure	Single Containment Attempt	Infinite Containment Attempts
T	$1/\lambda$	$1/\lambda$
G	c/λ	$(c/(1-c))(1/\lambda)$
G/T	c	$c/(1-c)$
**Unknown Hazard of Introduction Model**		
Measure	Single Containment Attempt	Infinite Containment Attempts
T	$E[1/\lambda]$	$E[1/\lambda]$
G	$cE[1/\lambda]$	$(c/(1-c))E[1/\lambda]$
G/T	c	$c/(1-c)$
**Avian Epidemic Model** *(Assuming Single Containment Attempt and c=100%)*		
	Exponential Increase	Linear Increase
T	$-e^{\lambda_0/r}Ei[-\lambda_0/r]/r$	$\exp\left(\frac{\lambda_0^2}{2r}\right)\sqrt{\frac{\pi}{2r}}\left(1-\text{erf}\left[\frac{\lambda_0}{\sqrt{2r}}\right]\right)$
G	$1/r+e^{\lambda_0/r}\lambda_0 Ei[-\lambda_0/r]/r^2$	$\frac{1}{2}\left(\frac{\lambda_0}{r}+\frac{\exp\left(\frac{\lambda_0^2}{2r}\right)\sqrt{\frac{\pi}{2}}(3r-\lambda_0^2)(1-\text{erf}[\lambda_0/\sqrt{2r}])}{r^{3/2}}\right)-\left(\exp\left(\frac{\lambda_0^2}{2r}\right)\sqrt{\frac{\pi}{2r}}\left(1-\text{erf}\left[\frac{\lambda_0}{\sqrt{2r}}\right]\right)\right)$
G/T	$-\frac{1}{\phi}-\frac{e^{-\frac{1}{\phi}}}{Ei[-\frac{1}{\phi}]}$	$\frac{1}{2}-\frac{x}{2}+\frac{\exp\left(-\frac{x}{2}\right)\sqrt{x}}{\sqrt{2\pi}\left(1-\text{erf}\left[\sqrt{\frac{x}{2}}\right]\right)}$

II. Additional Results

A. Alternate Method of Calculating Relative Gain Assuming the Constant Hazard Model

The results presented in the main text were derived using exponential-like right-skewed probability density functions for the gain *g* and the time to first introduction *t*. Because of this skewness, most realization of *g* and *t* may be smaller than their expected values *G* and *T*, respectively. Furthermore, the relative gain, calculated as *G*/*T*, may be different from the expected value of *g/t*. Below we calculate the skewness of the *g* and *t* PDFs and repeat the primary analysis from the main text using the median value of *g/t*. We show that the PDFs for *g* and *t* do show significant skewness. However, the results using E[*g*]/E[*t*] are very similar to those using the median *g/t*.

1. Calculating the Skewness Assuming the Constant Hazard Model

The skewness (γ) of $f_t(t)$ and $f_g(g)$ are calculated as

$$\gamma = \frac{\int (x - E[x])^3 f_x(x)dx}{\left(\int (x - E[x])^2 f_x(x)dx\right)^{3/2}}$$

where $E[x]$ is the mean and $f_x(x)$ is the PDF for random variable *x*.

The skewness for the time to first introduction is

$$\gamma = \frac{2\left(\varepsilon^3 + \rho(3 + \rho(3 + \rho))\right)}{\left(\varepsilon^2 + \rho(2 + \rho)\right)^{3/2}}$$

and the skewness for the gain is

$$\gamma = \frac{2\left(-3\varepsilon\rho - 3\varepsilon^2\rho^2 + \varepsilon^3(1 + 3\rho(1 + \rho)) + \rho(2 + \rho)(1 + \rho + \rho^2)(3 + \rho(3 + \rho))\right)}{\left(-2\varepsilon\rho + \varepsilon^2(1 + 2\rho) + \rho(2 + \rho)(2 + \rho(2 + \rho))\right)^{3/2}}$$

in terms of the relative magnitudes of the hazards.

Supporting Figure 2 shows plots of the skewness (γ) for the time to first introduction PDF (A and B) and the gain PDF (C and D). The horizontal axis is the effect of escalation ε and the vertical axis is the skewness γ. Each line shows the skewness at different values of ρ, the relative magnitude of the hazard of escalation. In A and C, ρ is 0, 0.1, 1 and 10 for the dotted, short dashed, long dashed and solid lines, respectively. In B and D, ρ is 100,1000,10000 and 100000 for the dotted, short dashed, long dashed and solid lines, respectively. The skewness for $f_t(t)$ is less than or equal to the skewness of an exponential PDF. The skewness for $f_g(g)$ is greater (and, for some parameter values, substantially greater) than or equal to the skewness of an exponential PDF. Thus the expected gain may be much larger than the gain for most realizations.

2. Calculating the Median *g/t* Assuming the Constant Hazard Model

ω is used to denote the ratio of the gain *g* to the time to first introduction *t*. We define the following transformations and their inverses:

$$\omega = p(g,t) = g/t \qquad g = m(\omega,s) = s$$
$$s = q(g,t) = g \qquad t = n(\omega,s) = s/\omega$$

The PDF of the ratio ω is the transformation of the bivariate probability density $f_{t,g}(t,g)$ to that for ω and *s*, integrated over the range of *s*:

$$f_\omega(\omega) = \int_0^\infty |J|\, f_{t,g}(t,g)\,ds$$

where $f_{t,g}(t,g)$ is equal to the product of $f_t(t)$ and $f_g(g)$ because *g* and *t* are independent random variables, and where $|J|$ is the absolute value of the determinant of the Jacobian matrix calculated as

$$|J| = \left| Det\begin{bmatrix} \partial m/\partial\omega & \partial n/\partial\omega \\ \partial m/\partial s & \partial n/\partial s \end{bmatrix} \right| = \left| Det\begin{bmatrix} 0 & -s/\omega^2 \\ 1 & 1/\omega \end{bmatrix} \right| = \left| \frac{s}{\omega^2} \right|.$$

Since *s* and ω^2 are positive, the Jacobian is always positive. The CDF for the ratio ω assuming the constant hazard model is

$$F_\omega(\omega) = \frac{\omega}{1+\omega}$$

with median value of 1. The results using the median ratio of the gain to time to first introduction are similar to the results using *G/T*.

B. Benefit of a Single Attempt Containment Policy Assuming the Stochastically Times Escalating Hazard of Introduction Model

The main text discusses the benefit of an infinite attempt containment policy with 50% success probability. Here we present additional results that show that a single attempt containment policy with 100% success probability provides a similar benefit.

Supporting Figure 3 shows the relative gain in time to a pandemic (*G/T*) assuming the escalating hazard of introduction model and assuming a single attempt containment policy with a 100% success probability. Each curve corresponds to a ten-fold increase in the magnitude of the escalation hazard relative to the initial hazard of introduction ($\rho=\lambda_E / \lambda_0$) ranging from 0.1 to 10 in A (short dashed, long dashed and solid lines), and from 100 to 100,000 in B (dotted, short dashed, long dashed and solid lines).

C. Benefit Assuming Deterministic Continuous Increase in the Hazard of Introduction Model

The main text discusses the benefit of a containment policy assuming a stochastically timed increase in the hazard of introduction. Here we show that the relative gain assuming any of several escalating hazard of introduction models is lower than that assuming a constant hazard of introduction. Specifically, we consider a deterministic increase in the hazard of introduction that is either exponential or linear.

Supporting Figure 4 shows the relative gain in time to a pandemic (*G/T*) as a function of the ratio (ϕ) of the rate of exponential increase in the virus's prevalence in birds (*r*) and the current hazard of introduction (λ_0) assuming a single attempt containment policy with 100% success probability. As with the escalating hazard of introduction model, the relative gain is at best one, but may be considerably lower, if the growth rate is much faster than the current hazard of introduction (ϕ>>1). Assuming that this increase is the result of an increase in the prevalence of the virus in bird populations, we expect that, at least in the early stages of spread in birds, this ratio would indeed be much larger than one: roughly speaking, when prevalence in birds is low, the prevalence in birds is likely to double before the first introduction of a human pandemic strain. Over time, the growing prevalence in birds will reduce this ratio by increasing the current hazard. However, the relative gain will never exceed 1.

Even with a sub-exponential increase in the rate, the relative gain may be relatively small. For example, if the number of avian cases increases linearly over time, the expected gain ranges from ½ to 1.

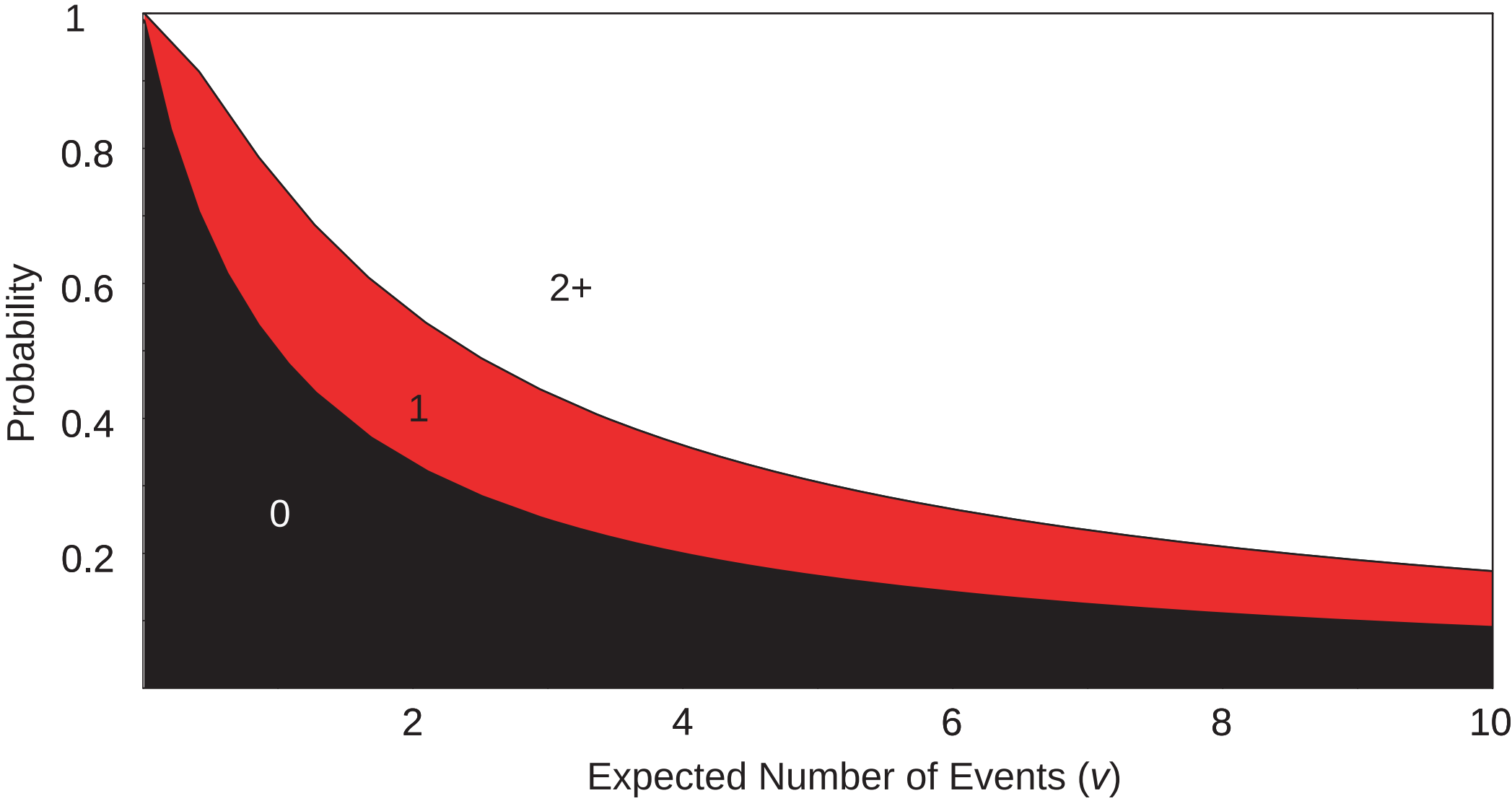

Supporting Figure 1

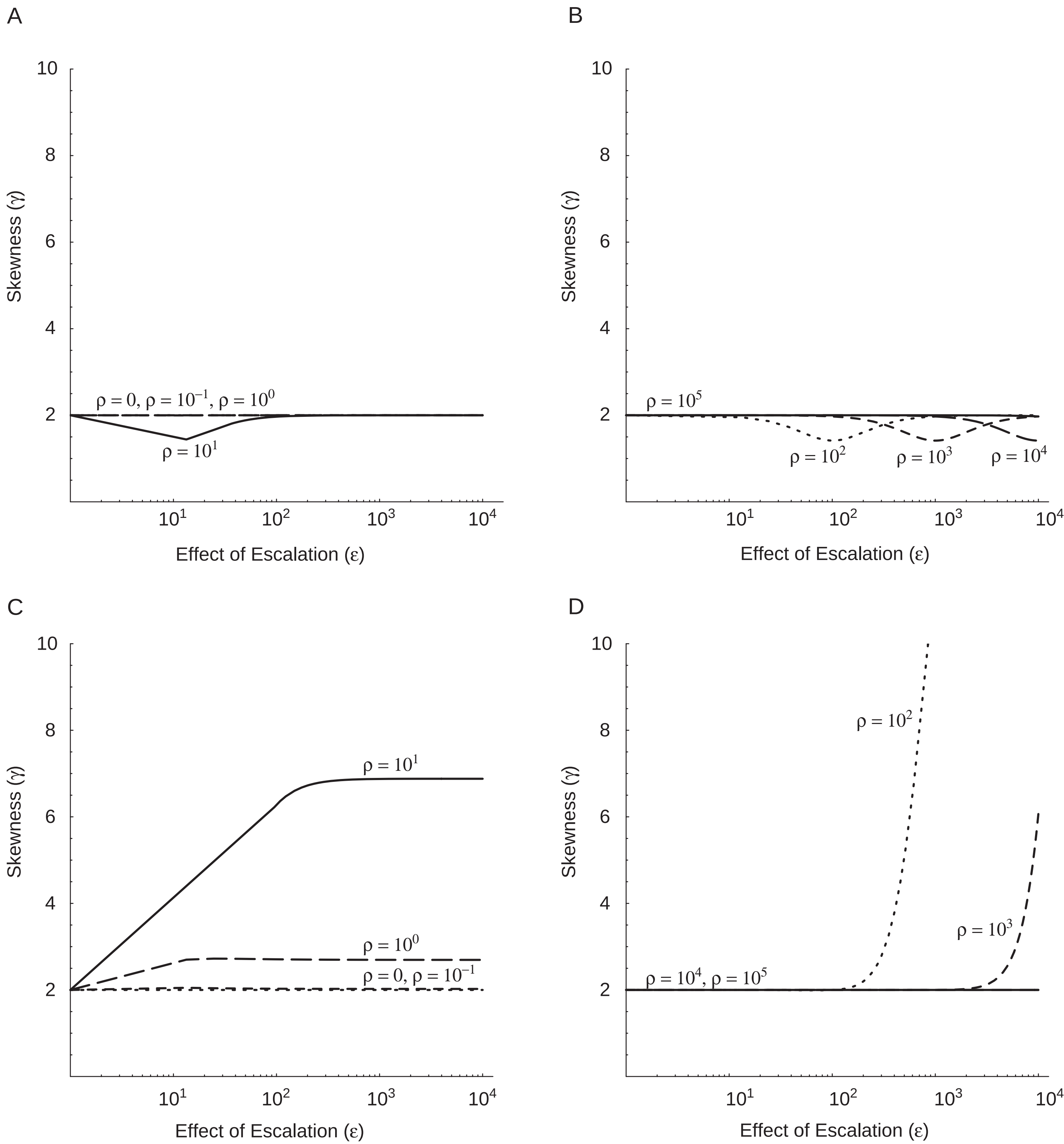

Supporting Figure 2

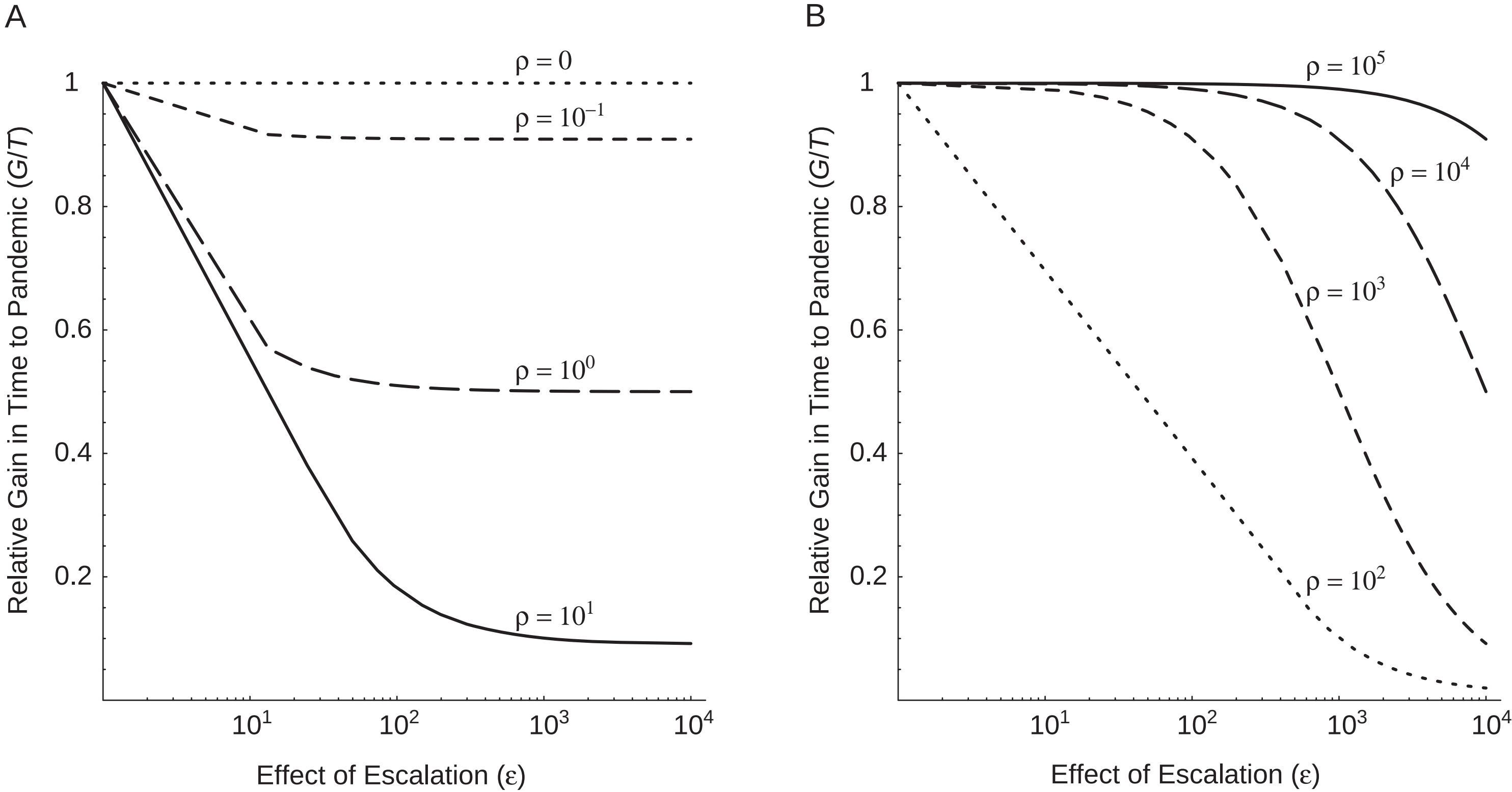

Supporting Figure 3

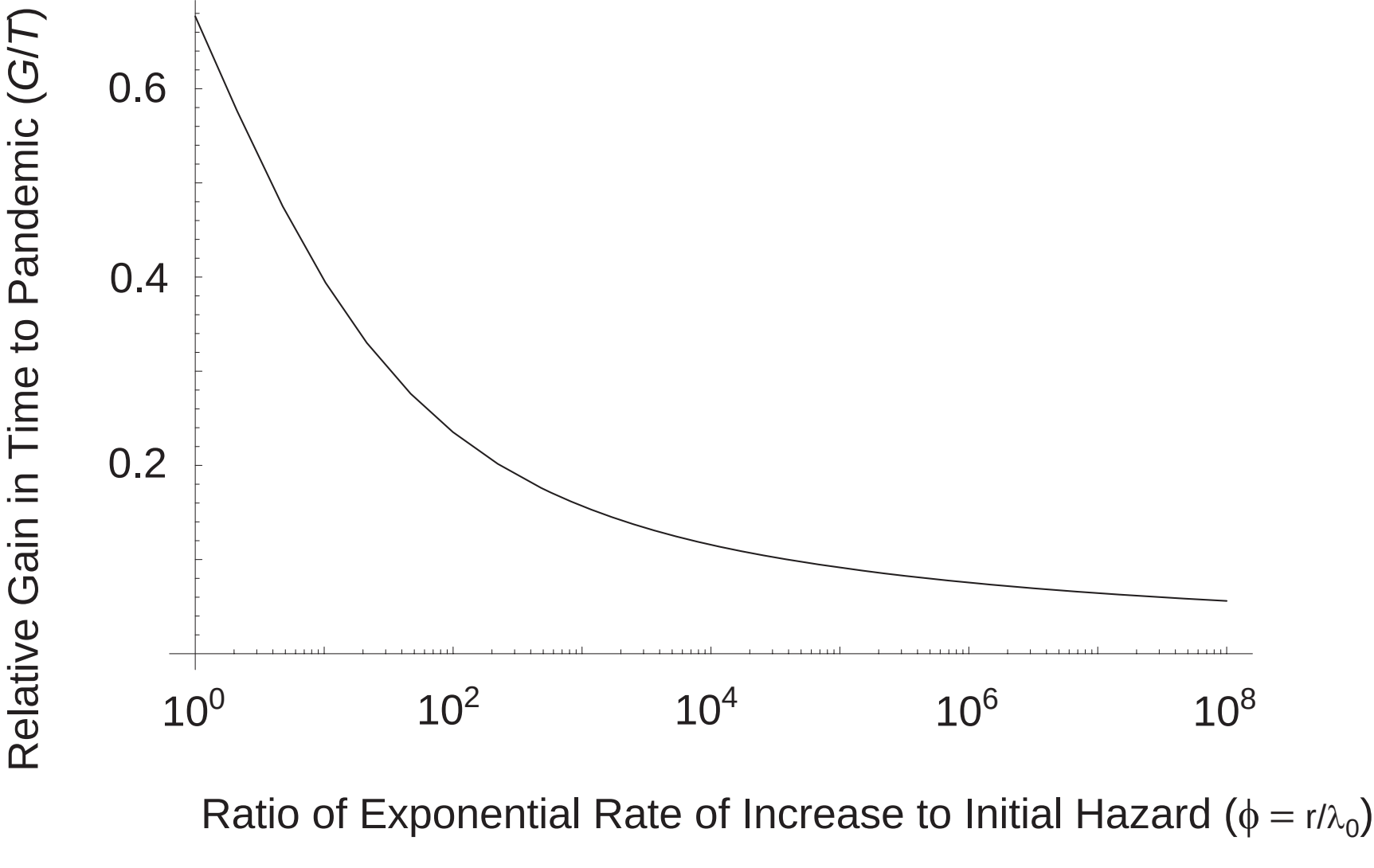

Supporting Figure 4
