# Supplementary material for: Pandemic Influenza: Risk of Multiple Introductions and the Need to Prepare for Them
Source: PLoS Med. 2006 Feb 21;3(6):e135. doi: 10.1371/journal.pmed.0030135 (PMC1370924; doi:10.1371/journal.pmed.0030135)
Supplement: Supplementary file 2 [file pmed.0030135.sd002.pdf]

Translation of the abstract ("Pandemic Influenza: Risk of Multiple Introductions and the Need to Prepare for Them") into Thai by Pantipa Rakkuson, Warakan Poomkumarn and Christina E. Mills

การระบาดของเชื้อไข้หวัดนกนี้อาจเกิดขึ้นได้ในระยะเริ่มต้นของการติดเชื้อหากเชื้อนั้นคือ H5N1 แต่หากมีการติดเชื้อที่จะแพร่ระบาดชนิดควบซ้อนหลายตัวก็อาจเป็นไปได้ยากขึ้น อย่างไรก็ดีการติดเชื้อระบาดชนิดควบซ้อนนี้มีโอกาสเกิดขึ้นได้โดยเฉพาะอย่างยิ่งเมื่ออยู่ในภาวะอัตราเสี่ยงต่อการระบาดสูงดังที่เป็นที่ยอมรับในขณะนี้ และเนื่องด้วยจำนวนชนิดของเชื้อที่สามารถควบซ้อนได้นี้มีมากขึ้นโอกาสที่การควบคุมการระบาดอย่างใดอย่างหนึ่งจะล้มเหลวก็ยิ่งมีมากตามไปด้วย เราได้ประเมินถึงข้อดีของยุทธการควบคุมการระบาดอันเกี่ยวเนื่องกับการเพิ่มขึ้นของระยะเวลากว่าจะถึงช่วงการระบาดภายใต้สมมติฐานที่แตกต่างกันเกี่ยวกับยุทธการการควบคุมการระบาดและอัตราของเชื้อที่สามารถควบซ้อน ซึ่งสมมติฐานที่สามารถเป็นไปได้มากที่สุดคือ นโยบายการควบคุมการระบาดอาจทำได้เพียงยืดระยะเวลาจนกว่าจะเกิดการระบาดขึ้นเป็นสองเท่า สรุปได้ว่าการวางแผนการควบคุมการระบาดนี้ควรรวมถึงแผนการที่ชัดเจนเกี่ยวกับวิธีการวัดผลที่รัดกุมในอันที่จะลดความเสี่ยงของเชื้อโรคที่อาจเกิดขึ้นในภายหลังซึ่งรวมถึงการฆ่าสัตว์ในวงกว้างเพื่อป้องกันการระบาดและการเปลี่ยนวิถีการทำเกษตรกรรมอันอาจเกิดขึ้นได้เมื่อมีการระบาดของเชื้อ
